# Graph neural networks in multi-stained pathological imaging: extended comparative analysis of Radiomic features

**DOI:** 10.1007/s11548-024-03277-x

**Published:** 2024-10-07

**Authors:** Luis Carlos Rivera Monroy, Leonhard Rist, Christian Ostalecki, Andreas Bauer, Julio Vera, Katharina Breininger, Andreas Maier

**Affiliations:** 1 Pattern Recognition Lab, Friedrich-Alexander-Universität Erlangen-Nürnberg, Erlangen, Germany; 2 Department of Dermatology, Universitätsklinikum Erlangen, Erlangen, Germany; 3 Department of Artificial Intelligence in Biomedical Engineering, FAU Erlangen-Nürnberg, Erlangen, Germany; 4 Center for AI and Data Science (CAIDAS), Universität Würzburg, Würzburg, Germany

**Keywords:** Radiomics, Multi-array imaging, Graph neural network, MELC, Histopathological analysis

## Abstract

****Purpose**:**

This study investigates the application of Radiomic features within graph neural networks (GNNs) for the classification of multiple-epitope-ligand cartography (MELC) pathology samples. It aims to enhance the diagnosis of often misdiagnosed skin diseases such as eczema, lymphoma, and melanoma. The novel contribution lies in integrating Radiomic features with GNNs and comparing their efficacy against traditional multi-stain profiles.

****Methods**:**

We utilized GNNs to process multiple pathological slides as cell-level graphs, comparing their performance with XGBoost and Random Forest classifiers. The analysis included two feature types: multi-stain profiles and Radiomic features. Dimensionality reduction techniques such as UMAP and t-SNE were applied to optimize the feature space, and graph connectivity was based on spatial and feature closeness.

****Results**:**

Integrating Radiomic features into spatially connected graphs significantly improved classification accuracy over traditional models. The application of UMAP further enhanced the performance of GNNs, particularly in classifying diseases with similar pathological features. The GNN model outperformed baseline methods, demonstrating its robustness in handling complex histopathological data.

****Conclusion**:**

Radiomic features processed through GNNs show significant promise for multi-disease classification, improving diagnostic accuracy. This study’s findings suggest that integrating advanced imaging analysis with graph-based modeling can lead to better diagnostic tools. Future research should expand these methods to a wider range of diseases to validate their generalizability and effectiveness.

## Introduction

The pursuit of accurate diagnostic methodologies in dermatology represents a critical aspect of contemporary medical research. This is particularly crucial in conditions such as melanoma, acknowledged as the most lethal form of skin cancer, where prognosis has traditionally depended on the expert analysis of clinical history and histopathology images [1]. The challenge is amplified by the heterogeneity of melanoma subtypes, necessitating detailed lesion characterization. Albrecht et al. [2] highlight the critical importance of feature standardization in mathematical models for an accurate assessment of melanoma, underscoring the complexity of this disease. To address these challenges, emerging graph-based methods target an integration of biological parameters with diverse domain features, offering a more universal approach to skin cancer diagnosis.

Similarly, the diagnostic journey in other dermatological conditions, like eczema, presents its own set of challenges. As a chronic inflammatory skin disease prevalent in both children and adults, eczema demands swift and accurate diagnosis due to its high incidence. However, it is frequently misdiagnosed as other skin conditions, highlighting a significant diagnostic challenge in dermatology [3]. The variability in staining protocols across different clinical centers further complicates the use of computational models in distinguishing eczema from other diseases [4]. This underscores the need for the development of more adaptable and universally applicable diagnostic models.

The complexities of dermatological diagnostics are further exemplified in the diagnosis of lymphoma using histopathology slides. Syrykh et al. [5] discuss how the histopathological diagnosis of lymphomas heavily relies on expert analysis and is influenced by the technical processing of tissue sections, leading to potentially time-consuming procedures and a heightened risk of misdiagnosis. Additionally, a systematic review by Bai et al. revealed significant heterogeneity in the diagnostic performance of artificial intelligence (AI) algorithms in detecting lymphoma from medical imaging [6]. This variability in diagnostic effectiveness underscores the need for further development and understanding of AI-assisted methods for their integration into clinical practice.

Building upon our previous work, where we explored ways of describing pathology samples on a cellular level using graphs and classification tasks for melanoma MELC samples [7] and discussed the impact of Radiomic features on node classification of graphs compared to encoding cell-wise average intensity for each staining agent (multi-stain profiling) [8], this study further explores the supervised classification of multiple-epitope-ligand cartography (MELC) pathology samples. It extends the comparative analysis of two distinct feature types: multi-stain profiles from multiplex digital imaging and Radiomic features. These features are analyzed within both traditional machine learning frameworks and graph neural networks. Notably, this extended research investigates not only the cell-wise classification capabilities but also the slide-wide disease classification using these features. Additionally, dimensionality reduction techniques, such as uniform manifold approximation and projection (UMAP) and t-distributed stochastic neighbor embedding (t-SNE), are employed on the features encoded in the graph nodes to assess their impact on classification accuracy, especially in conjunction with graph neural networks (GNNs). This approach not only builds upon our previous findings but also offers new insights into pathology-assisted diagnostics.

In this work, we first introduce the histopathological data used (Sect. "Datasets"), selecting a set of challenging and relatively common diseases: eczema and lymphoma on the one hand, melanoma on the other. Next, we briefly discuss the types of features used to characterize the data on the digital slides (Sect. "Exploring radiomic features through dimensionality reduction techniques"). Following this, we describe the graph creation process (Sect. Data-driven graph formation"), where this specific data structure represents the problem at two levels: disease-wise (whole slide of the pathology sample) and cell-wise (each cell in the sample is modeled independently). Additionally, we briefly describe the selected Graph Neural Network architecture (Sect. "Graph neural network architecture"), highlighting its main strengths and why it was chosen for this study. This work also explores the influence of dimensionality reduction techniques on reducing complexity and their impact on overall model performance. Using graphs allows the classification problem to be modeled at two different levels. In the experimental design section (Sect. "Experimental design"), we describe how we set up the data and compare the trained classifiers with baseline models to evaluate the capabilities of graph-driven approaches for the set of diseases. Finally, we present the classification results at both the disease and cell levels (Sect. "Results")highlighting that our approach, which combines the modeling of pathological samples using graphs and Radiomic features, provides positive results for both classification tasks. We then discuss these results in more detail and outline possible future work (Sect. "Discussion").

## Materials and methods

**Fig. 1:**
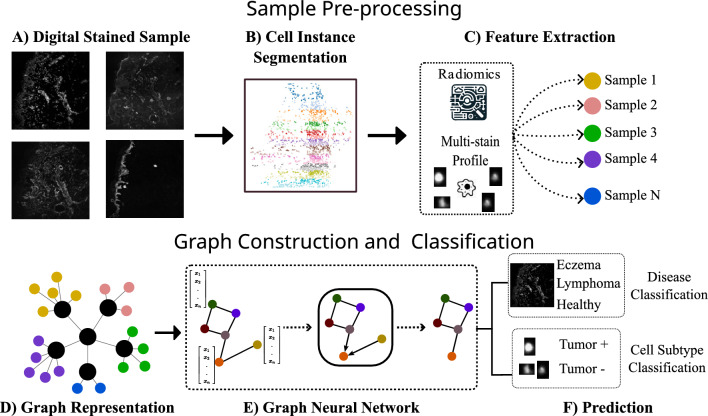
Overview of the methodological approach. **A** Preparation of MELC samples and annotations. **B** Extraction of cell instances using Cellpose. **C** Feature extraction step involving the extraction of either stain profiles or Radiomic features. **D** Construction of the graph structure. **E** Training of the graph neural network, specifically using the Grand+ model. **F** Utilization of the trained network for prediction purposes

### Datasets

In this research, we extend our previous methodology [7, 8], which initially focused on a dataset for cell-wise classification involving suspected melanoma tissue samples. This dataset included specimens from 37 cases: 20 confirmed melanoma instances and 17 healthy tissue samples. Each sample was processed using the MELC protocol, a technique involving stages such as incubation with specific affinity reagents, application of fluoroscope-coupled antibodies, capturing detailed images via immunofluorescence microscopy, and the bleaching of staining agents. This approach allows the collection and analysis of the response for up to 100 different antigens [9]. Continuing from the established setup, we ensured that each sample in this study was treated with a comprehensive range of 80–90 different staining agents, enabling an in-depth analysis of each specimen.

Following this thorough staining process, the resulting images were digitally captured, each boasting a high resolution of 0.45 $\upmu$m/pixel and dimensions of $2018 \times 2018$ pixels (Fig. 1A). In instances where tumors were identified, meticulous expert segmentation was performed by a medical professional. This approach not only ensures consistency with the methodologies outlined in our previous work but also facilitates a direct and detailed comparison and follow-up of the findings.

For the expanded scope of this study, we delve into the efficacy of Radiomic-extracted features, comparing these against the multi-stain profiles created by the iterative application of different reagents. Our exploration occurs on two distinct analytical levels. The first level is a whole sample multi-class classification task, where samples are categorized into one of three groups: healthy, eczema, or lymphoma. This dataset includes a diverse range of samples: 27 from lymphoma patients, 24 from eczema patients, and 25 healthy control samples. All were collected from distinct patients at the diagnosis stage, prior to any treatment. The second analytical level focuses on cell-wise classification within a set of 20 melanoma cases, compared to 17 healthy tissue samples. Here, the primary objective is to discern cells associated with melanoma from those in healthy regions.

### Exploring Radiomic features through dimensionality reduction techniques

Incorporating Radiomic attributes into graph-based methods significantly improves the precision of cell classification in histopathological examinations, as we previously explored in [8] for melanoma samples in the context of cell-wise binary classification tasks. These attributes encompass primary intensity statistics, morphological descriptors, and textural elements. Critical in this context are the texture characteristics derived from the Gray-Level Co-occurrence Matrix (GLCM) and the Gray-Level Run Length Matrix (GLRLM), which offer comprehensive cellular insights [10]. These Radiomic features were extracted using the pyradiomics package [11]. GLCM evaluates the spatial correlations of pixel intensities, while GLRLM measures the continuity of pixel intensity runs in an image. As attributes in graph nodes, these features significantly mitigate challenges like sensitivity to initialization and local minima in graph optimization [12]. To compare the effectiveness of these features, we will contrast them with those used in previous approaches, where each node had a feature vector composed of the intensity values for each of the staining agents used, referred to as the “multi-stain profile.” This vector representation is obtained from the average value per stain from the mask generated from the cell instance segmentation (Fig. 1B), which is done using Cellpose to extract cell instances [13] (Fig. 1C).

Additionally, it is important to note that the Radiomic features are extracted from stains related to the specific disease. In our previous work [7], we thoroughly examined a broad set of stains. The selection of stains in that study was based on clinical prior knowledge, focusing on those with a high correlation to the specific disease being studied. For this study, we have narrowed our focus to a selection of the most relevant stains from the available pool, ensuring that the Radiomic features we extract are highly pertinent to the disease under investigation.

Dimensionality reduction approaches such as uniform manifold approximation and projection (UMAP) and t-distributed stochastic neighbor embedding (t-SNE) are instrumental in this framework. These methodologies are applied to the extracted set of features, condensing the high-dimensional datasets into lower-dimensional representations that are computationally manageable. This step is crucial as it prepares the feature set for subsequent use in training the graph neural networks (GNNs). By reducing the dimensionality of the data, these techniques enable the creation of intricate yet efficient neighborhood graphs [14, 15], which form the basis for the graph models in our study. This synergy of advanced imaging analysis with graph-based modeling substantially elevates the accuracy of cell classification and provides profound insights into biological architectures in histopathological images [16]. Consequently, the graph models infused with Radiomic data become vital instruments for discerning cellular patterns, thereby augmenting diagnostic accuracy and contributing significantly to histopathological research.

### Data-driven graph formation

In our previous work, we discussed the feasibility of using graph-based models to encapsulate MELC-derived profiles, providing a potential pipeline to classify cell instances in melanoma regions [7]. Moreover, we investigated the impact of Radiomic features on this task when employed as input features for the graph [8]. This research generated encouraging outcomes, which inspired the current work to explore the application of these methods in classifying histopathological samples of diseases that are challenging to detect early or are often misdiagnosed.

The problem we address in this study is the classification of histopathological samples at both the cell and sample levels. We formulate this as a graph-based classification problem, where each sample or cell is represented as a node, and the relationships between them (based on spatial proximity or feature similarity) are captured by the edges. The goal is to use the graph structure to predict the class labels (e.g., healthy, eczema, lymphoma) for each node, whether it represents an individual cell or an entire sample.

The foundation of graph construction lies in defining the nodes and edges. For nodes, we consider cell instances extracted using the Cellpose pretrained model for cell segmentation [13]. In other words, each node represents a cell instance found on the whole slides. As for the edges, we established a fixed neighborhood of 14 cells to ensure a fair comparison between two different connectivity criteria, which will be tested separately. The first criterion connects cells that are spatially closest, as detailed by Wolf et al. [17]. The second criterion links cells with high mutual information, indicative of similar features, similar to the approach described by Palla et al. [18]. The size of the neighborhood was selected to balance performance and model complexity, allowing us to effectively compare the impact of spatial closeness and feature similarity on graph connectivity. This process is depicted in the pipeline shown in Fig. 1D.

Additionally, we introduced “connector nodes,” which are artificial nodes created to facilitate information flow across different parts of the graph. These nodes serve as central hubs to connect different samples of the disease and ensure that information can travel from one end of the graph to the other. This approach allows for a more comprehensive integration of data from various samples.

**Fig. 2:**
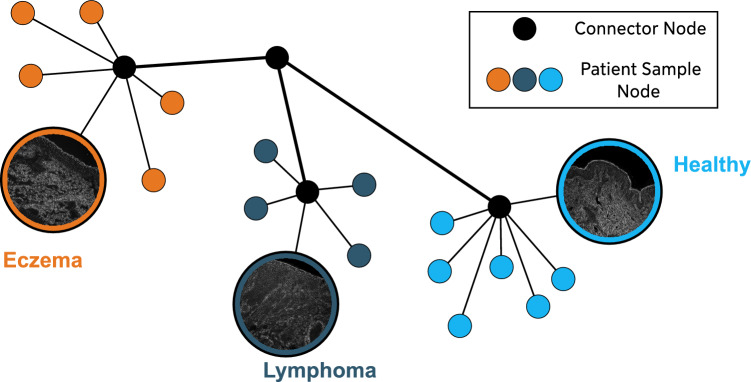
Detailed illustration of the graph construction for disease classification. In this multi-class case, each disease is linked to a central connector node. Additionally, individual samples to each disease are also connected to their respective disease-specific connector nodes. This structure effectively represents the relationship between different diseases and their corresponding samples, forming the foundation for the classification process

**Fig. 3:**
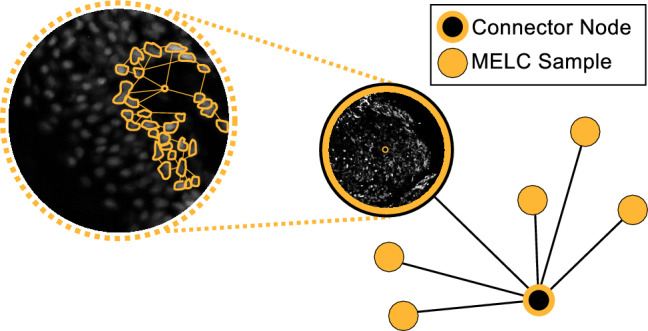
Graph construction for binary classification of melanoma cells in MELC samples. This figure illustrates the graph formation where each melanoma MELC sample is connected to a unique connector node. These connector nodes are in turn linked to a central connector node, representing the overarching classification structure. This arrangement facilitates the analysis and binary classification of individual cell instances, distinguishing whether they are within or outside the melanoma region

In these representations, a typical sample contained approximately 2,000 cells. In the multi-class disease classification, we constructed a graph comprising 27,320 nodes and 191,240 edges. A schematic of how this graph representation looks can be seen in Fig. 2. For the cell-wise analysis of melanoma samples, we constructed a graph of 40,500 nodes and 202,500 edges. A schematic of how the cell-wise graph representation looks can be seen in Fig. 3.

### Graph neural network architecture

Developed by Feng et al., the GRAND+ architecture is an evolution of their original GRAND model and represents a significant advancement in the field of graph neural networks [19]. At its core, GRAND+ is a transformer-based architecture specifically designed to process graph-structured data. It operates by propagating node features across the graph through a series of message-passing layers, which allow each node to aggregate information from its neighbors. This process is guided by the attention mechanism inherent in transformer architectures, enabling the model to focus on the most relevant nodes during training.

In addition to the basic transformer framework, GRAND+ incorporates several advanced techniques to improve performance. One of its key features is the incorporation of consistency regularization, which helps the model maintain stable predictions across different input views. This regularization is particularly beneficial in ensuring that the model generalizes well across different datasets, which may have varying levels of data quality and annotation consistency.

For a detailed explanation of the GRAND+ architecture, including its specific components and training algorithm, we refer the reader to the original work by Feng et al. [20]. In our study, we leverage this architecture due to its demonstrated ability to handle the complexities of our specific problem domain, which involves classifying both entire histopathological samples (disease-level classification) and individual cells within those samples (cell-level classification). The architecture’s ability to generalize effectively in these scenarios, where different conditions are represented within the same graph, is a key factor in its selection.

Our choice of GRAND+ over other state-of-the-art graph neural network architectures, such as graph convolutional networks (GCNs) and graph attention networks (GATs), was driven by its superior performance in supervised learning tasks. While GCNs and GATs are effective in many applications, they did not offer the same level of generalization and adaptability needed for our specific tasks. The transformer-based approach of GRAND+ allows it to manage the diverse and variable nature of our datasets more effectively, making it the optimal choice for our objectives.

### Experimental design

In this study, we adhered to the performance evaluation methodology established in our previous works, allocating the data into training, validation, and test sets. The data were distributed to ensure balanced representation of samples across each segment of the dataset, as shown in Table 1. Hyperparameter optimization was systematically conducted on the validation set using a Bayesian search strategy, implemented through the Weights and Biases platform [21].

**Table 1:** Dataset splits for different classification tasks

Task	Dataset	Split		
		Train	Validation	Test
Disease classification	Eczema	11	3	10
	Lymphoma	13	3	11
	Healthy	11	3	11
Cell subtype classification	Melanoma	14	2	4
	Healthy	10	3	4

For baseline comparisons, we utilized XGBoost and Random Forest, both of which are widely recognized in the field of machine learning for their efficacy in handling tabular data. The training of the graph neural network (GNN) was conducted in accordance with the guidelines provided by the original authors and followed a similar approach to hyperparameter optimization [20].

The primary objective of our analysis is to evaluate the effectiveness of two distinct types of features: staining profiles derived from multi-array imaging and features generated by Radiomics. These Radiomic features include first-order intensity statistics, shape-based descriptors, and intricate texture features. The purpose of this comparative study is to shed light on the relative strengths and potential applications of these feature sets within our machine learning models. An illustrative overview of the proposed pipeline is presented in Fig. 1.

#### Disease classification

In the initial set of experiments, our work investigates the framework’s capability for multi-class classification of histopathology samples into three critical categories: eczema, lymphoma, and healthy tissue. This approach involves constructing a comprehensive graph as depicted in Fig. 2, which integrates the diseases into a single general graph. Here, each sample and disease possesses a central node. During training, these central nodes facilitate learning the representation initially from the sample and subsequently from the disease itself. In the testing phase, the sample of interest is connected to the central nodes of all diseases, and edge weight values are predicted for each. The classification is determined by selecting the disease that exhibits the highest edge weight value with the sample, indicating the greatest similarity (Fig. 1F).

#### Cell subtype classification

For the second set of experiments, we investigate the model’s effectiveness at the cell-wise level by conducting binary classification to determine if a cell is located within or outside the melanoma region (Fig. 1F). Figure 3 presents an overview of the graph structure employed. During the training phase, the graph learns representations at the cell level by treating each cell instance as a node. These nodes are connected to a central node within each sample, which is then linked to a global node that connects all samples. This design ensures that the model learns cellular representations not just from individual cases, but from the collective patterns across multiple samples. The goal is to train the graph neural network (GNN) to effectively classify each cell (node) based on these learned representations.

In the testing phase, when the model is exposed to unseen data, the graph utilizes the learned representations to classify the new nodes (cells) as either positive (within the melanoma region) or negative (outside the region). This process demonstrates how the model generalizes the information learned during training to make accurate predictions on new samples.

Additionally, we explored how the GNN behaves when exposed to two different sets of features: Radiomic features, which are highly descriptive and capture intricate details of cell morphology and texture, and multi-stain profiles, which are derived from the chemical properties of staining agents and provide a more natural representation of cellular characteristics. By comparing these feature sets, we aim to assess the GNN’s ability to generalize and classify cells accurately under varying types of input data.

**Fig. 4:**
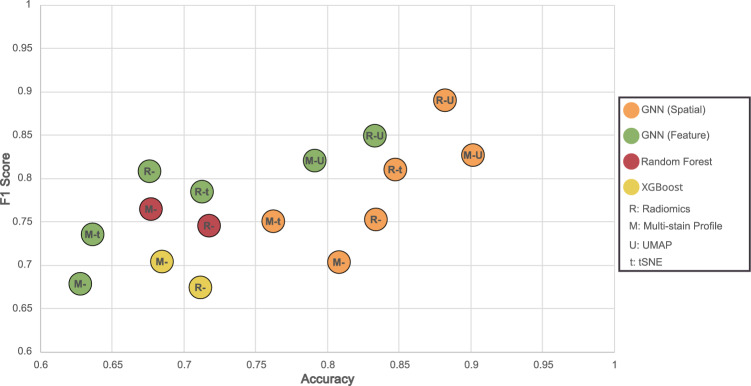
Comparative analysis in multi-class classification. This scatter plot contrasts multi-stain profiles and Radiomics across four methods, incorporating dimensionality reduction for GNN approaches. The *x*-axis shows accuracy, and the *y*-axis depicts the weighted F1 score, illustrating the performance balance in classifying eczema, lymphoma, and healthy samples

## Results

In this section, we present the findings from our two distinct sets of experiments conducted on MELC samples. The first experiment revolves around the multi-class classification of histopathological samples, distinguishing between eczema, lymphoma, and healthy control tissues. The second experiment investigates the binary classification of cell instances, determining whether they are part of the melanoma tumor region. These results provide valuable insights into the effectiveness of our proposed methodologies in handling complex classification tasks in medical imaging.

### Disease classification results

In the results of our first experiment, as depicted in Fig. 4, we present the qualitative outcomes from the training of a multi-class graph neural network classifier. This classifier is tasked with categorizing new samples into eczema, lymphoma, or healthy tissue. The scatter plot in the figure compares the performance of baseline methods, namely XGBoost and Random Forest, with the graph neural network approach, focusing on metrics such as the weighted F1 score and accuracy. The weighted F1 score is particularly relevant in this context because it accounts for class imbalance by averaging the F1 scores of each class according to their prevalence, ensuring that performance is measured accurately across all categories. Accuracy is also a crucial metric as it provides a straightforward measure of the overall correctness of the model’s predictions. Together, these metrics offer a comprehensive evaluation of the model’s performance across the different classes.

**Fig. 5:**
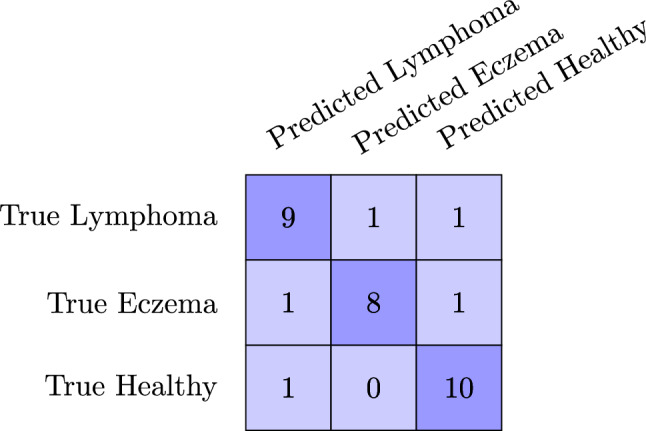
Confusion matrix for the multi-class classification problem: the matrix shows the distribution of true and predicted classes for lymphoma, eczema, and healthy samples for the best model (graph with Radiomic features and UMAP reduction)

Our analysis extends to differentiating between two connectivity criteria in the graph neural network, as outlined in Sect. "Data-driven graph formation". The results demonstrate that the GNN model utilizing spatial criteria for defining connectivity outperforms the one using feature similarity as the sole criterion. Alongside this, we investigated the impact of dimensionality reduction techniques, specifically UMAP and t-SNE (as detailed in Sect. "Exploring radiomic features through dimensionality reduction techniques"), on the training and testing phases of GNNs. These techniques have been previously shown to significantly influence outcomes. The findings indicate that while baseline models maintain stable performance across various feature types, the GNN methods display enhanced performance when using Radiomic features compared to multi-stain profiling, regardless of the connectivity criteria. Additionally, the application of dimensionality reduction not only streamlines training but also significantly elevates GNN performance, particularly evident with UMAP, where both Radiomic and multi-stain profiling features see marked improvements in their performance This improvement is clearly reflected in the confusion matrix shown in Fig. 5, where we observe an accurate classification across eczema, lymphoma, and healthy samples, demonstrating the model’s superior capability to differentiate between the classes.

### Cell subtype classification results

**Fig. 6:**
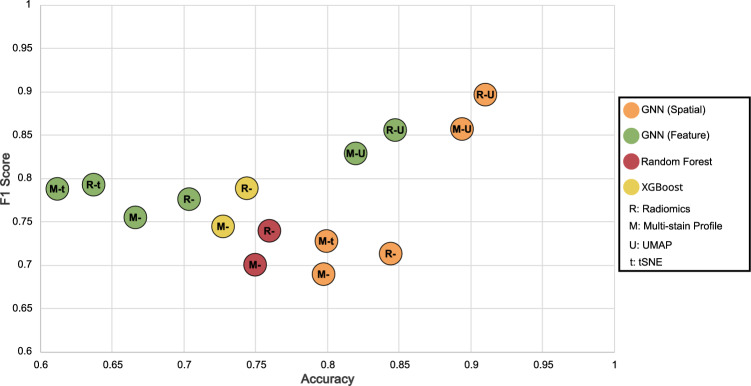
Binary classification results in melanoma cell analysis. This scatter plot compares multi-stain profiles and Radiomics for different methods, with accuracy on the *x*-axis and weighted F1 score on the *y*-axis, demonstrating their effectiveness in melanoma cell analysis

Figure 6 in our second set of experiments illustrates a scatter plot detailing the quantitative results from the binary classification training focused on cell instances within or outside the melanoma tumor region. The evaluation of performance was based on the average accuracy and weighted F1 score for each disease. As outlined in the previous section, XGBoost and Random Forest served as baseline methods. Both spatial and feature connectivity criteria, described in Sect. "Data-driven graph formation", were considered. The baseline methods demonstrated remarkably similar performances, showing no significant difference when using either Radiomics or multi-stain profiling as input features. Notably, Radiomics consistently yielded positive results as the input feature, surpassing multi-stain profiling. Furthermore, the spatial connectivity criterion led to superior overall performance in GNNs compared to the feature similarity criterion for GNN construction and training.

## Discussion

As we conclude this study, detailed in Sect. "Results", we reflect on the significant advancements made in the classification of MELC pathology samples, particularly for diseases such as eczema, lymphoma, and melanoma, as well as in cell-wise classification within melanoma tumors.

The multi-class classifier, as explored in Sect. "Disease classification results", demonstrates the aptitude of graph neural networks (GNNs) for disease classification in pathology samples that are often challenging to distinguish due to their similarities. This finding is consistent with related research that suggests the utility of graph representations in capturing complex biological relationships [22]. Our results particularly underscore the superior performance of spatial connectivity criteria in such classifications, echoing the findings of Gao et al. [23] and Mason et al. [24] regarding the importance of spatial relationships in biological systems modeled using graphs.

Additionally, our investigation reaffirms the beneficial impact of dimensionality reduction on GNNs, aligning with previous work and studies discussing the advantages of integrating methods like UMAP with GNNs [25]. Radiomics, as an extracted feature set, has also proven its worth in the classification of pathological slides in multi-disease studies, offering a valuable perspective on the representation of pathology samples and their cellular compositions.

The integration of Radiomic features and graph representation offers a promising framework for assisted diagnosis. However, this approach does call for further exploration in more diverse diseases and a broader array of samples to adequately test its generalization capabilities.

Turning to the binary classification results described in Sect. "Cell subtype classification results", the updated GNN model (Grand+) has shown enhanced training efficiency and adaptability in the domain of medical imaging. These results validate the approach for precise melanoma assessment, offering valuable tools for clinical research in disease prognosis and progression.

Concluding our analysis, we note:

- The proposed frameworks exhibit strong performance in MELC data analysis, showcasing their potential in medical research.
- There are some limitations that persist due to the limited number of MELC samples, indicating a need for broader studies to ascertain the generalizability of this framework.
- Future directions include delving into the explainability of models and features, particularly understanding which Radiomic features contribute most significantly to performance enhancements.
- Additionally, we aim to enhance the graph representation mechanism, enabling it to learn more effectively from various diseases, thus reducing misclassification rates.

This study, therefore, not only contributes to the current understanding of disease classification using advanced computational methods but also opens avenues for more detailed and comprehensive research in the field.

## Data Availability

Data will be made available on request.

## Code availability

Code and extensive description of the parameters of the best performing model are available on the GitLab Repository.
